# Chimeric Antigen Receptor-Modified T Cells for Solid Tumors: Challenges and Prospects

**DOI:** 10.1155/2016/3850839

**Published:** 2016-02-21

**Authors:** Yelei Guo, Yao Wang, Weidong Han

**Affiliations:** Department of Immunology, Institute of Basic Medicine, Chinese PLA General Hospital, Beijing 100853, China

## Abstract

Recent studies have highlighted the successes of chimeric antigen receptor-modified T- (CART-) cell-based therapy for B-cell malignancies, and early phase clinical trials have been launched in recent years. The few published clinical studies of CART cells in solid tumors have addressed safety and feasibility, but the clinical outcome data are limited. Although antitumor effects were confirmed* in vitro* and in animal models, CART-cell-based therapy still faces several challenges when directed towards solid tumors, and it has been difficult to achieve the desired outcomes in clinical practice. Many studies have struggled to improve the clinical responses to and benefits of CART-cell treatment of solid tumors. In this review, the status quo of CART cells and their clinical applications for solid tumors will be summarized first. Importantly, we will suggest improvements that could increase the therapeutic effectiveness of CART cells for solid tumors and their future clinical applications. These interventions will make treatment with CART cells an effective and routine therapy for solid tumors.

## 1. Introduction

Recently, chimeric antigen receptor-modified T- (CART-) cell-based therapy, an innovative approach to tumor treatment, was demonstrated to potentially exhibit MHC-independent antitumor effects. These cells could directly recognize tumor cells by genetic modification to express a chimeric antigen receptor (CAR), and they were activated to exhibit a durable persistence* in vivo* through the T-cell activation endodomain with costimulatory signaling molecules [1, 2]. After two decades of preclinical research and clinical trials, the safety and feasibility of CART-cell-based therapy have been confirmed, and unprecedented clinical results have been obtained in hematological malignancies [3–5]. For example, several groups have reported clinical trials with anti-CD19 CART cells in which favorable clinical efficacy resulted from the specific recognition and eradication of CD19-positive tumor cells [3, 4, 6]. These clinical studies indicate that CART-cell therapy can produce clinical responses in patients with advanced hematological malignancies.

**Figure 1:**
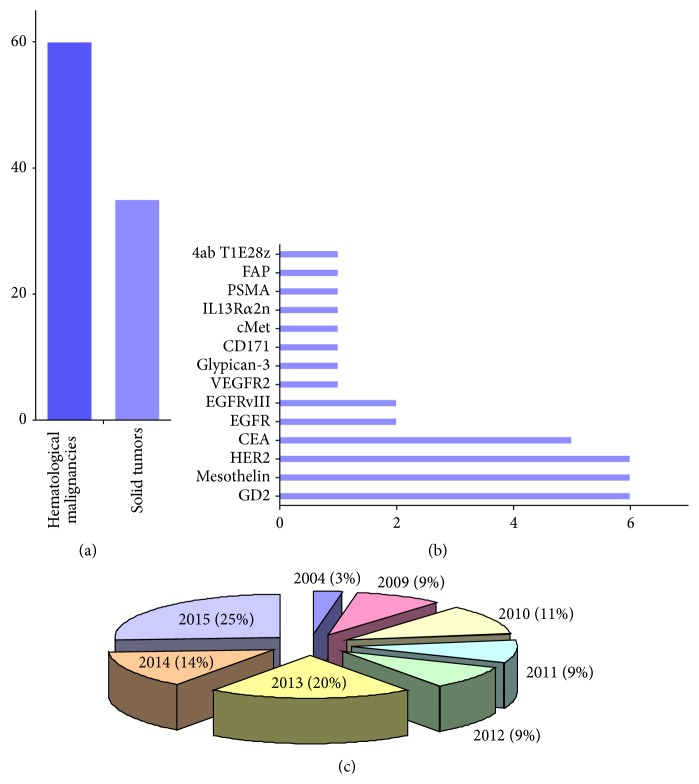
Current status of clinical trials of chimeric antigen receptor-modified T (CART) cells in malignancies. These data were searched on 15 June, 2015, from the website ClinicalTrials.gov (http://www.clinicaltrials.gov). The key phrases “chimeric antigen receptor-modified T cells”, “chimeric antigen receptor”, “CART”, and “CAR” were used. (a) Comparison of the number of registered CART-cell trials for solid tumors and hematological malignancies on the ClinicalTrials.gov website. (b) The registered solid tumor targets for CART cells on the ClinicalTrials.gov website. EGFR: epidermal growth factor receptor; FAP: fibroblast activation protein; PSMA: prostate-specific membrane antigen; VEGFR2: vascular endothelial growth factor receptor 2. (c) Proportion of annual registered numbers of CART cells in solid tumors on the ClinicalTrials.gov website.

The clinical studies of CART cells for solid tumors have begun recently. Up to date, eleven studies of CART-cell therapy for solid tumors have been conducted in the past decade (Table 1), and thirty-five clinical trials for various solid tumors are listed at ClinicalTrials.gov (http://www.clinicaltrials.gov) (Figure 1). The registered numbers of clinical trials increase annually, and a range of tumor antigens, including CEA, mesothelin, HER2, and GD2, are being targeted for various solid tumors.

In preclinical studies, antitumor efficacy of CART cells has been confirmed* in vitro* and in animal experiments; however, the clinical outcomes in recent studies of CART cells treating solid tumors remain marginal, even though the safety and feasibility have been established [7–9]. Recently, several studies have attempted to search efficient approaches to improve the effectiveness of CART cells for solid tumors. In this review, we discuss the main challenges that impede the development of favorable clinical responses in solid tumors, and we suggest improvements for future clinical applications of CART cells.

## 2. A Concise History of the Clinical Applications of CART Cells in Solid Tumors

CAR redirected T-cell-based therapy has emerged as a promising strategy for malignant diseases since the first report by Gross et al. in 1989 [10]. In the past two decades, several studies have demonstrated encouraging clinical outcomes in patients with B-cell malignancies that are treated by CART cells, and the results from these studies indicated that CART cells could produce clinical responses in other types of cancer [3, 4, 6]. Theoretically, CART-cell therapy could be curative for solid tumors if the genetically modified T cells encountered the tumor cells* in vivo*. Accordingly, the development of CART cells for solid tumors is imperative in the clinic. Nevertheless, there are few reports of successful clinical studies of solid tumors that are treated with CART cells.

Thus far, CART-cell-based therapy has been tested against several types of solid tumors, including ovarian cancer, neuroblastoma, colon cancer, and mesothelioma (Table 1) [11–14]. In the first clinical study, three patients with metastatic renal cell carcinoma who were administered CART cells specific for CAIX developed liver toxicity [7]. And a further trial of 12 patients treated with anti-CAIX CART cells is still ongoing to assess the safety of the cells [8]. Further initial reports demonstrated encouraging outcomes in 30 patients with neuroblastoma treated with CART-GD2 cells [5, 12]. A clinical study of neuroblastoma from another center used CD171-specific CART cells and indicated some evidence of antitumor efficacy [9]. Importantly, these studies show that CART-cell therapy is safe for patients with advanced solid tumors, but the use of first-generation CART cells and their limited survival may account for the lack of a spectacular clinical response.

To enhance the persistence of CART cells and improve the clinical outcome in solid tumors, costimulators, such as CD28, 4-1BB, and OX40, were integrated into the fusion CAR protein [13, 15, 16]. In one case report, a patient with colon cancer that metastasized to the lungs and liver, who received conditioning lymphodepletion and was treated with 10^10^ third-generation ERBB2-specific CD28.4-1BB.*ζ*-CART cells combined with IL-2, developed acute respiratory distress syndrome and died five days after the treatment [13]. In another study, sarcoma patients treated with up to 10^8^/m^2^ second-generation CART cells encoding a HER2.CD28.*ζ*-CAR without conditioning chemotherapy or administration of IL-2 experienced no toxicity, but the antitumor effect was limited [15]. Several other clinical studies with CEA- and mesothelin-specific second-generation CART cells for solid tumors have been reported recently, and the safety and efficacy of this cell-based therapy have been confirmed [14, 16, 17].

Taken together, the clinical experience with CART-cell therapy for solid tumors suggests that several factors, including the tumor antigens, costimulatory molecules, CART-cell development process, and conditioning therapies, likely contributed to the different clinical outcomes. Thus, several urgent issues need to be resolved to improve the safety and clinical responses of CART cells for patients with solid tumors.

## 3. Potential Challenges for CART-Cell Treatment of Solid Tumors in the Clinic

Although CART-cell-based therapy has been shown to be a potential treatment strategy for few solid tumors [14, 15, 17], the challenges to this strategy that affect safety and clinical outcomes should be addressed. The current critical issues are discussed in the following.

### 3.1. The Screening of Solid Tumor Target Antigens

Preclinical studies on CART cells that are specific for many different tumor antigens expressed on solid tumors have been conducted and have shown antitumor effects [18, 19]. To date, numerous potential solid tumor target antigens have been explored for CART-cell-based therapy (Table 2), but unfortunately, few antigens are uniquely specific for solid tumors. A major concern of CART cells in solid tumor treatments is ensuring the effective elimination of tumor cells while avoiding the off-tumor/on-target toxicity that caused when these T cells attack healthy tissues. Experience indicates several principles that should be observed to overcome this problem: (1) preferred selection of specific tumor antigens and (2) selection of tumor targets based on their expression level and frequency on tumor and normal tissues [20, 21]. The density of tumor antigen expression can also affect the selection of CART-cell targets [22].

In addition, it is well known that tumor-associated antigens can be divided into two groups, including mutated antigens (also called neoantigens) and “self-” antigens such as tissue/lineage antigens, developmental antigens, and overexpressed antigens [23]. Most of the recent studies have indicated that cancer immunotherapies have remained focused on recognizing “self-” antigens; however, only few immunotherapies target neoantigens [24, 25]. Neoantigens, short 8 to 12 amino acid peptides that are known to be created by cancer cell genomes mutations, can be rapidly identified by high-throughput next-generation sequencing (NGS) in several cancers, including melanoma, ovarian cancer, and cholangiocarcinoma [19–26]. In contrast to “self-” antigens that are expressed on tumor and normal cells, neoantigens are only found in tumor cells, showing accurate specific targets for cancer immunotherapy to reduce the risk for autoimmune disease, for example, a splice variant of the epidermal growth factor receptor (EGFRvIII) [26, 27]. Based on their specific features, recent clinical evidence has confirmed that neoantigens are the best potential targets for adoptive T-cell therapy with least possible toxicity [28]. Therefore, it is reasonable that a strategy using CART cells that specifically target neoantigens is the best potential therapeutic treatment for cancers without severe target-mediated toxicity.

### 3.2. Optimizing the Affinity of the CAR

The affinity of CAR is also important for its antitumor effect and target-mediated toxicity. The relationship among the CAR affinity and density and tumor antigen density could impact the effector function of CART cells. Low affinity was more effective than high-affinity CAR under conditions when the levels of CAR were limiting, whereas no significant difference was observed on the variance of CAR affinity on conditions of high levels of CAR expression [29]. In addition, high-affinity CAR did not increase the activity of T cells against target tumor cells compared with low CAR affinity, and the high-affinity CAR distinguished less well between tumor cells with high or low levels of antigen expression, whereas low affinity CAR showed negligible responses to tumor antigens expressed at low or undetectable levels, but they were highly reactive to the tumor cells that overexpressed antigen [30, 31]. A recent study on the sensitivity of CAR to EGFR density indicated that CAR with reduced affinity could render CART cells able to distinguish tumor from normal tissues, and their antitumor effects were decreased along with the reduced density of EGFR [32]. On the basis of the careful conclusions from previous studies on solid tumors, it is possible to select the reduced affinity of CAR to avoid the off-tumor/on-target toxicity when target antigens are overexpressed on tumor cells and expressed at low levels in normal tissues. However, for highly specific tumor antigens, high-affinity CAR should be considered to prevent tumor escape when tumor cells express a low level of antigens.

### 3.3. The Source of the Single-Chain Fragment of the Variable Region Antibody (scFv)

Most existing studies have derived the scFv components of the chimeric receptor from mouse monoclonal antibodies [11, 33]. Although this construct only contains the variable regions of the mouse monoclonal antibody, a human anti-mouse antibody by the recipient could, after cell infusion, block the interaction between CAR and the target tumor antigen to inhibit the antitumor effect of the CART cells. The use of humanized scFvs or scFvs derived from human monoclonal antibodies for CAR will solve this issue. Advances in biotechnology will expand the prospects for humanized scFvs for CART-cell-based therapy for solid tumors.

### 3.4. Costimulatory Molecules

To improve the expansion of CART cells in solid tumors, costimulatory molecules, including CD28 and 4-1BB, have also been incorporated in the CAR gene by several groups, with increased persistence* in vivo* [14, 15, 17]. Recent studies indicated that CD28 can accelerate T-cell expansion, leading to T-cell exhaustion and reduced cell persistence compared with the 4-1BB domain [34]. Additionally, it has been reported that 4-1BB is superior to CD28 costimulation because 4-1BB preferentially promotes the expansion of memory T cells, whereas CD28 expands naïve T cells [35]. However, other studies showed that there was no any clear superiority for either CD28- or 4-1BB-based CART cells. For example, no significantly different cytotoxicity* in vitro* and* in vivo* was observed on CART cells with either a CD28 or 4-1BB costimulator, although CD28-based CART cells produced higher IL-2, IL-6, and IFN-gamma levels [36]. Other studies showed that the expansion and antitumor cytotoxicity by CD28- and 4-1BB-based CART cells were similar [37]. In addition, Hombach et al. demonstrated that CD28-CART cells were superior to CD28-OX40-CART cells because the CD28-OX40 super-costimulation increased activation-induced cell death (AICD) and reduced the cells' antitumor function [38]. In contrast, some studies indicated that the CAR gene containing two costimulators, such as CD28 and 4-1BB, yielded improved T-cell survival and cytotoxicity compared with a single co-stimulator [37, 39]. After careful consideration, these studies indicate that the choice of costimulatory molecules affects the therapeutic response, but it remains unclear whether any costimulatory molecule is superior to another [40, 41]. Therefore, more attempts to develop CAR with different costimulatory molecules are urgently needed to further explore the therapeutic outcomes* in vitro* and* in vivo*.

Here, some suggestions for the choice of costimulatory molecules will be delineated for solid tumors. For solid tumors, the migration to the tumor sites is a prerequisite for CART cells to play an antitumor efficacy. Once breaking through the tumor microenvironment and making contact with target cells, CART cells need to undergo rapid expansion to have an antitumor function, while avoiding inhibition by the tumor environment. In clinical trials, for example, CD28 was associated with faster expansion than 4-1BB costimulation, and multiple cycles of infusion could overcome the shorter persistence of CD28-based CART cells in solid tumors.

### 3.5. The Optimal Processing of T Cells Specific for Solid Tumors

The response of solid tumors to CART cells in clinical studies has been limited [7–9]. These suboptimal outcomes could reflect the use of first-generation CART cells with a low ability to persist. Costimulation by integrating CD28 or 4-1BB into CAR molecules can improve the persistence of CART cells* in vivo* [42–44]. Moreover, the differentiation states (e.g., naïve T cells) and replicative frequencies of T cells could be key to achieving better clinical outcomes [45–47]. Previous studies have indicated that the stimulus and cytokine environment in the cell culture process can determine the T-cell differentiation state. For example, IL-7, IL-15, and IL-21 could slow T-cell differentiation [48, 49], whereas activation by soluble anti-CD3 and CD28 monoclonal antibodies achieved optimal T-cell differentiation [50]. Activation by soluble anti-CD3 and CD28 monoclonal antibodies in the presence of IL-15 and IL-21 enhanced T cells with a naïve phenotype and with a lower proportion of CD4^+^CD25^+^CD127^−^ expression [47].

Trafficking to and accumulating in the tumor sites are prerequisites for CART cells to play an antitumor efficacy, particularly for solid tumors. Nevertheless, CART cells cannot easily contact with target tumor cells due to the tumor microenvironment, resulting in the inability of these infused cells to fully activate and proliferate. T-cell migration to tumor sites requires integrins, chemokines, and chemokine receptors [51, 52]. However, cell culture* in vitro* and genetic modification could cause the loss of chemokine receptors, possibly resulting in CART cells being unable to localize accurately to the tumor tissues [53]. In previous studies, chemokine receptors, such as CXCR2 and CCR4, were genetically modified to be expressed on T cells to enhance their homing and antitumor activity [54, 55]. Therefore, the forced expression of integrins, chemokines, and chemokine receptors on CART cells could improve their migration ability and promote their antitumor activity.

### 3.6. Preconditioning Therapy

Immunotherapy is a promising and efficient approach to cancer treatment. Basic research and clinical studies indicate that only a fraction of patients achieve durable clinical responses after immunotherapy. The immune system is highly important for maintaining a balance between protection from tumor development and the promotion of tumor growth, whereas tumor cells can escape the immune system leading to cancer progression that is facilitated by the tumor microenvironment when the balance is destroyed [56, 57]. The microenvironment of solid tumors has been reported to interfere with the desired clinical outcome through multiple networks of cellular interactions, which could create immune tolerance and negate immunotherapies, including CART-cell-based therapy. The tumor microenvironment is extremely complex and contributes to tumorigenesis and metastasis by limiting immune responses to cancer cells and preventing the eradication of tumors [58]. Interference with immune cell infiltration, activation, and proliferation in the tumor microenvironment can ultimately facilitate tumor development, metastasis, and resistance to therapy. Therefore, strategies to counteract the tumor microenvironment and to enhance antitumor effects are urgently needed.

Immunosuppressive cells (e.g., regulatory T lymphocytes, Tregs) can be induced to accumulate in tumor site by the tumor microenvironment, playing an essential role in tumorigenesis [59]. Preconditioning therapy to remove Tregs can effectively enhance the antitumor effects of CART cells for solid tumors. Fortunately, chemotherapy can make the tumor microenvironment highly permissive for antitumor immunity [60]. Chemotherapeutic agents, such as cyclophosphamide, docetaxel, and pemetrexed, could impair Treg function and enhance the host's immunity in clinical studies [61–63]. Other strategies have been explored to reduce Tregs. For example, denileukin diftitox, an IL-2-diphtheria toxin fusion protein, directly killed Tregs through selective targeting of CD25 in preclinical cancer models [64]. A high-dose of IL-2 could downregulate the level of Tregs, at least in the periphery [65].

In addition, previous studies demonstrated that lymphodepleting chemotherapy preconditioning could enhance the antitumor efficacy of tumor-infiltrating lymphocytes [66]. Lymphodepletion creates an appropriate “lymphoid space” for the proliferation of adoptive infused immune cells. Additionally, lymphodepleting conditioning can improve the expansion and persistence of CART cells in solid tumor patients.

To the best of our knowledge, radiotherapy commonly induces tumor cell death through cell stress by altering cellular survival, and by apoptosis pathways and cell cycle regulatory mechanisms [67]. However, preclinical studies have also indicated that radiotherapy can make tumor cells more immunogenic by several mechanisms [68–70]. First, radiotherapy can make the tumor microenvironment more susceptible to attack by immune cells [71]. Second, tumor antigen expression is increased after local treatment by radiotherapy [72]. Third, radiotherapy could induce intratumoral dendritic cells expressing chemokines that attract immune cells into tumor sites [73, 74]. Finally, Fas, ICAM-1, and NKG2D ligands were upregulated on tumor cells after radiotherapy [75–77]. Based on this information, radiotherapy could play a role in enhancing adaptive antitumor effects, in addition to promoting the regression of tumors. Therefore, the antisolid tumor effects of CART cells could be enhanced by radiotherapy.

## 4. Strategy of CART Cells Specific for Tumor Stroma

Immunotherapy aims to improve the clinical antitumor response of cancer patients. Nevertheless, for many immunotherapies, the tumor microenvironment is the major barrier to an antitumor response [78]. Tumor stroma, a composition of the tumor microenvironment, could support tumor growth and resistance to therapy by the following mechanisms [67, 79–84]: (1) blocking therapeutic agents that attack tumor cells; (2) producing growth factors, chemokines, and matrix that could support tumor growth, invasion, and angiogenesis; (3) expressing inhibitory surface molecules such as programmed death-1 ligand (PD-L1) and PD-L2, producing factors to attract Tregs, myeloid-derived suppressor cells, and macrophages, and secreting factors to regulate T-cell functions to create an immunosuppressive milieu to inhibit immune cell function; and (4) the mechanisms of tumorigenesis that are supported by stroma coexisting among a variety of stromal cell types.

The most recent clinical studies see CART cells as attacking tumor cells. However, there can be limitations to the use of CART cells that are specific for solid tumors as, for example, tumor stroma, which could create bias towards an undesirable clinical response, compared with the considerable success in the treatment of hematologic malignancies. CART cells might not activate and proliferate well due to the tumor stroma inhibiting immune cells from making contact with tumor cells. Therefore, a strategy to disrupt the tumor stroma could improve the antitumor function of immunotherapy. CART cells that are specific for tumor stroma could promote the treatment of a broad spectrum of solid tumors.

To date, four attempts using CART cells that are specific for the fibroblast activation protein (FAP) that is highly expressed in cancer-associated stroma cells have been reported in animal models [85–88]. Antitumor activity was observed after CART-cell administration in these studies, although adverse events, such as on-target toxicity, were also observed because FAP is also expressed on normal tissues, including pancreas, lung, and bone marrow [85, 88]. The on-target/off-tumor toxicity occurred because the scFv that targeted mouse stroma caused the CART cells to attack normal mouse stroma cells [85, 88]. In contrast, other studies employing CART cells derived from human T cells and a scFv that targeted mouse or mouse/human stroma had no adverse events [86, 87]. More importantly, the antitumor efficacy of the endogenous immune cell antitumor response was augmented by the CART-cell infusion; the CART cells lost their antitumor effect in immunodeficient mice [88].

Based on the data from the preclinical studies, CART cells that target tumor stroma could be candidates for solid tumor treatment in the future. However, several issues should be addressed before their clinical application: (1) the selection of the tumor stroma cell antigen; (2) the development of protocols to augment the antitumor effect for CART cells by combination with other immunotherapies, such as CART cells that are specific for tumor cells; and (3) the concern for potential adverse events such as on-target/off-tumor toxicity.

## 5. Novel Concept of CAR Design for the Precision Treatment in Solid Tumors

Tumor antigens are important to activate CART cells to induce immune activity against tumor cells. Nevertheless, solid tumor cells typically express highly heterogeneous tumor-associated antigens, rendering them able to escape detection by the immune system [89]. Only few antigens are tumor-specific for the treatment of solid tumors using CART cells. Although recent clinical studies indicated that CART cells were safe and feasible for solid tumors, on-target/off-tumor toxicity remains the main concern impacting their clinical application. Accordingly, novel concepts of CAR design for solid tumor precision treatment have been explored to enhance the on-tumor specificity. Recently, several studies indicated that bispecific CAR design could improve the tumor cell specificity and limit the target-mediated toxicity of CART cells. Contrary to conventional CART cells that only target a single antigen, bispecific CART cells can recognize multiple antigens by expressing two CARs on genetically modified T cells. For example, in preclinical models, T cells expressing two CAR molecules specific for PSMA and PMCA specifically targeted prostate cancer cells, and they were only activated in the presence of both antigens, not by either alone [90]. Another concept of bispecific CAR design uses a negative signal to enhance the tumor specificity: in one example a cytotoxic T lymphocyte antigen-4- (CTLA-4-) or programmed death-1- (PD-1-) based antigen-specific inhibitory CAR (iCAR) was designed to preemptively constrain T cells' responses [91]. These T cells selectively limited their cytokine secretion, cytotoxicity, and proliferation in response to normal tissues on which the iCAR was present. Bispecific CART cells, expressing a CAR and an iCAR specific for an antigen present on normal tissues, could avoid a CART-cell-mediated attack on normal tissues, consequently enhancing tumor specificity [91, 92]. In addition, the tandem CAR (TanCAR) design, which is also a bispecific CAR, can recognize each antigen and improve the activation and effective function when it encounters both antigens simultaneously using a single CAR molecule with two antigen recognition moieties that are joined in tandem [93]. The novel concept of CAR design to genetically modify T cells to target multiple tumor antigens could avoid the risk of immune escape [94]. This approach can also protect normal tissues by increasing the tumor specificity of CART cells. Ultimately, we must optimize the testing of bispecific CART cells to ensure their safety and efficacy before their clinical application for solid tumors.

In addition, to reverse on-target/off-tumor toxicity, several attempts to encode suicide genes in CART cells have shown that this adverse event can be irreversibly prevented through the selective destruction of the infused genetically modified T cells [95–97]. The addition of suicide genes to CART cells could ensure their safety for solid tumor treatments, avoiding unwanted and severe adverse events and increasing on-tumor specificity.

**Figure 2:**
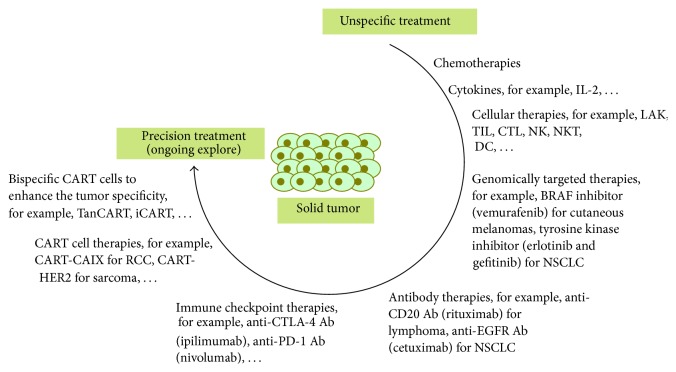
Development of precision treatment for solid tumor. Ab: antibody; CTL: cytotoxic T cells; CTLA-4: cytotoxic T lymphocyte-associated antigen-4; DC: dendritic cells; iCART: inhibitory signal-based antigen-specific CART cells; IL-2: interleukin-2; LAK: lymphokine-activated killer cells; NK: natural killer cells; NKT: natural killer T cells; NSCLC: non-small-cell lung cancer; PD-1: programmed death-1; TanCART: tandem CART cells; TIL: tumor-infiltrating lymphocytes.

The precision treatment for solid tumors is improving more rapidly due to advances in biotechnology development (Figure 2). Recent advances in CART-cell-based therapy are currently being translated from the laboratory to the clinic. Novel concepts of CAR design could ensure the clinical application of CART cells for solid tumors with enhanced tumor specificity. Coupled with individual and diversified interventions (such as chemotherapies and vaccines), the precision of CART cells could provide great promise for the treatment of solid malignant patients in the future.

## 6. Combinatorial CART-Cell Therapy to Improve Clinical Benefit in Solid Tumors

The ultimate goal of cancer therapy is to be curative, including CART-cell immunotherapy. However, for solid tumors, the microenvironment is the major barrier to treatment with immunotherapy. It is necessary to develop a potent product to prevent the suppressive function of the solid tumor microenvironment to enhance the antitumor activity of CART-cell therapy. To the best of our knowledge, it is well known that solid tumors can create a complex microenvironment to defend against an attack from the immune system. For example, the antitumor effect of T cells can be inhibited by expressing PD-1 when it interacts with its ligands PD-L1 and PD-L2 that are expressed on tumor cells and/or stroma cells [98, 99]. Most* in vitro* and preclinical data have indicated that the blockade of the interaction between PD-1 and PD-L1 or PD-L2 provides a potentially promising approach for cancer immunotherapy by improving the response of T cells [99, 100]. Several clinical studies of anti-PD-1 monoclonal antibody have demonstrated the safety and activity for patients with advanced solid tumors, such as melanoma, non-small-cell lung cancer, and renal cell cancer [101, 102]. In addition, a phase I clinical study showed evidence for an antitumor effect of anti-PD-L1 antibody against advanced solid tumors [103]. It is promising that two antibodies against PD-1 (pembrolizumab and nivolumab) have been approved by the U.S. Food and Drug Administration in 2014 [58]. Therefore, exploration of CART cells combined with PD-1/PD-L1-specific antibodies is expected to increase the antitumor effect in solid tumors.

Several negative regulators other than PD-1 have been identified and reported to inhibit the response of T cells to attack against tumors, for example, CTLA-4, T-cell immunoglobulin and mucin-containing protein 3 (TIM-3), lymphocyte-activated gene-3 (LAG-3), T-cell immunoreceptor with Ig and ITIM domains (TIGIT), B and T lymphocyte attenuator (BTLA), and V-domain Ig suppressor of T-cell activation (VISTA) [104]. The continued development of CART-cell therapy combined with inhibitors of these negative regulators could improve their clinical benefit in solid tumors.

## 7. CART Cells as a Primary Strategy for Treating Solid Tumors

Due to economic and medical technological factors, most cancer patients are diagnosed at an advanced disease stage. The strategies for treating patients with advanced solid malignant diseases mainly include surgery, chemotherapy, radiotherapy, targeted therapy, and supportive care, but cancers generally relapse or become refractory, denying patients their best opportunity for treatment. Recent studies indicated that adoptive cell transfer treatments can stimulate and improve the function of the immune system and overcome chemotherapy resistance [105, 106]. Nevertheless, patients are often first treated by traditional approaches rather than by the adoptive transfer of immune cells.

CART-cell treatment as a primary strategy needs to be implemented urgently to increase the therapeutic benefit for patients with solid tumors. Although experience with the adoptive transfer of CART cells to treat solid tumors remains limited, technological improvements will enhance clinical responses in the future. Several tasks should be addressed, including (1) careful screening of patients to ensure that they have the specific tumor target to reduce the risk of on-target/off-tumor adverse event; (2) suggesting CART-cell therapy as a primary strategy for patients and clinical researchers, alone or in combination with other therapies; (3) establishing the benefit of using CART cells as a first treatment; (4) monitoring and resolving the toxicities in these strategies; and (5) analyzing the clinical response compared with other therapies. In addition, to improve the clinical response and standardize the procedures, large-scale, controlled, grouped, and multiple-center clinical trials are of particular importance to implement. On this basis, the treatment of solid tumors by CART cells as a primary strategy can be extended.

## 8. Conclusions and Perspectives

Efforts to treat solid tumors with CART cells are ongoing. Considering the recent studies together, treatment with CART cells has been shown to be safe and is thus potential promising for the treatment of solid tumors. However, none of these CART-cell-based strategies has been superior to the existing options, and a number of the challenges and limitations mentioned above must be resolved to ensure better patient benefit and to extend this treatment approach. Based on previous studies, the safety and clinical responses are still the main exploring focuses in the future. CART cells combined with other therapies, such as chemotherapy, radiotherapy, and PD-1/PD-L1 antibodies, will also be relevant. The best clinical responses can be achieved through careful preparation of patients, CART cells and doses, preconditioning regimens, and follow-up treatments. In addition, CART cells will likely be commercialized to increase their convenience and flexibility for patients with solid tumors, or even other malignancies, using streamlined, centralized, and large-scale generation of CART cells from uniform cell sources. These interventions will make treatment with CART cells an effective and routine therapy for solid tumors. In conclusion, although more work is needed to meet the challenges, treatment with CART cells has a significant potential to improve clinical responses in solid tumors.

## Figures and Tables

**Table 1:** Recent published clinical studies on CART cells specific for solid tumor antigens.

Antigen	CAR	Gene transfer	Cancer	Case number	Clinical outcome	Time	Reference
HER2	ScFv-CD28-CD3*ζ*	Retrovirus	HER2-positive sarcoma	19	1 PR, 4 SD	2015	[15]
CEA	ScFv-CD28-CD3*ζ*	Retrovirus	CEA+ liver metastases	8	1 SD, 5 DOD	2015	[17]
Mesothelin	ScFv-4-1BB-TCR*ζ*	Electrotransfer	Mesothelioma	2	1 PR, 1 SD	2014	[14]
Mesothelin	ScFv-4-1BB-TCR*ζ*	Electrotransfer	Mesothelioma	1	1 PR	2013	[16]
CAIX	ScFv-Fc*ε*RI*γ*	Retrovirus	CAIX+ metastatic RCC	12	NED	2013	[8]
GD2	ScFv-CD3*ζ*	Retrovirus	Neuroblastoma	19	3 CR, 1 PR	2011	[5]
ERBB2^*∗*^	ScFv-CD28-4-1BB-CD3*ζ*	Gamma-retrovirus	Colon cancer	1	Dead	2010	[13]
GD2	ScFv-CD3*ζ*	Retrovirus	Neuroblastoma	11	1 CR, 2 SD, 2 tumor necrosis	2008	[12]
CD171	ScFv-CD3*ζ*	Electrotransfer	Neuroblastoma	10	1 PR	2007	[9]
FR	ScFv-Fc*ε*RI*γ*	Retrovirus	Ovarian cancer	8	NED	2006	[11]
CAIX	ScFv-Fc*ε*RI*γ*	Retrovirus	CAIX+ metastatic RCC	3	NED	2006	[7]

CAIX: carboxy-anhydrase-IX; CEA: carcinoembryonic antigen; CR: complete response; DOD: dead of disease; FR: folate receptor; HER2: human epidermal growth factor receptor 2; NED: no evidence of disease; PR: partial response; RCC: renal cell carcinoma; ScFv: single chain fragment of variable region antibody; SD: stable disease.

^*∗*^HER2/neu.

**Table 2:** Potential solid tumor targets for CART cell-based therapy.

Antigen	Cancer
CD44v7/8	Cervical carcinoma
DNAM-1	Prostate carcinoma
EGP-40	Colorectal cancer
EpCAM	Prostate cancer
FBP	Ovarian cancer
FR	Rhabdomyosarcoma
GD3	Melanoma
VEGFR2	Tumor neovasculature
LMP-1	PVR and nectin-2 expressing solid tumors
MUC1	Breast, ovary
PSCA	Melanoma, synovial cell sarcoma

DNAM-1: DNAX accessory molecule-1; EGP-40: epithelial glycoprotein-40; EpCAM: epithelial cell adhesion molecule; FBP: folate-binding protein; LMP-1: latent membrane protein 1; MUC1: mucin 1; PSCA: prostate stem cell antigen.
